# RKIP Pleiotropic Activities in Cancer and Inflammatory Diseases: Role in Immunity

**DOI:** 10.3390/cancers13246247

**Published:** 2021-12-13

**Authors:** Roni Touboul, Stavroula Baritaki, Apostolos Zaravinos, Benjamin Bonavida

**Affiliations:** 1 Department of Microbiology, Immunology & Molecular Genetics, David Geffen School of Medicine at UCLA, University of California at Los Angeles, Los Angeles, CA 90095, USA; rtouboul@g.ucla.edu; 2 Laboratory of Experimental Oncology, Division of Surgery, School of Medicine, University of Crete, Heraklion, 71003 Crete, Greece; baritaks@uoc.gr; 3 Department of Life Sciences, School of Sciences, European University Cyprus, Nicosia 2404, Cyprus; a.zaravinos@euc.ac.cy; 4 Basic and Translational Cancer Research Center (BTCRC), Cancer Genetics, Genomics and Systems Biology Laboratory, Nicosia 1516, Cyprus

**Keywords:** RKIP, T cells, cancer, immunosuppression, immunotherapy, autoimmunity

## Abstract

**Simple Summary:**

The human body consists of tissues and organs formed by cells. In each cell there is a switch that allows the cell to divide or not. In contrast, cancer cells have their switch on which allow them to divide and invade other sites leading to death. Over two decades ago, Doctor Kam Yeung, University of Toledo, Ohio, has identified a factor (RKIP) that is responsible for the on/off switch which functions normally in healthy tissues but is inactive or absent in cancers. Since this early discovery, many additional properties have been ascribed to RKIP including its role in inhibiting cancer metastasis and resistance to therapeutics and its role in modulating the normal immune response. This review describes all of the above functions of RKIP and suggesting therapeutics to induce RKIP in cancers to inhibit their growth and metastases as well as inhibit its activity to treat non-cancerous inflammatory diseases.

**Abstract:**

Several gene products play pivotal roles in the induction of inflammation and the progression of cancer. The Raf kinase inhibitory protein (RKIP) is a cytosolic protein that exerts pleiotropic activities in such conditions, and thus regulates oncogenesis and immune-mediated diseases through its deregulation. Herein, we review the general properties of RKIP, including its: (i) molecular structure; (ii) involvement in various cell signaling pathways (i.e., inhibition of the Raf/MEK/ERK pathway; the NF-kB pathway; GRK-2 or the STAT-3 pathway; as well as regulation of the GSK3Beta signaling; and the spindle checkpoints); (iii) regulation of RKIP expression; (iv) expression’s effects on oncogenesis; (v) role in the regulation of the immune system to diseases (i.e., RKIP regulation of T cell functions; the secretion of cytokines and immune mediators, apoptosis, immune check point inhibitors and RKIP involvement in inflammatory diseases); and (vi) bioinformatic analysis between normal and malignant tissues, as well as across various immune-related cells. Overall, the regulation of RKIP in different cancers and inflammatory diseases suggest that it can be used as a potential therapeutic target in the treatment of these diseases.

## 1. Introduction

The Raf-1 kinase inhibitory protein (RKIP), also referred to as PEBP-1 or PBP, is a member of the phosphatidylethanolamine-binding protein (PEBP) family that was originally isolated from the bovine brain [1]. It is a small, cytosolic protein [2] with wide expression in the tissues of various mammalian species, including monkeys, rats, chickens, and humans [1,3,4,5,6,7]. RKIP, as a very dynamic protein with a flexible pocket loop, exists in a number of states to enhance its functional switch [8,9]. The RKIP molecule appears to have pleiotropic activities on multiple signaling pathways, thus critically affecting cellular processes associated with their activation [10,11,12].

Yeung et al., (2000) first identified RKIP as a physiological endogenous inhibitor of the Raf-1/MEK/ERK signaling pathway [13] via direct binding to all three MAP kinases. The binding of RKIP to both Raf-1 and MEK inhibits their phosphorylation and activation, thus leading to downstream suppression of the Raf-1-induced signaling and activity of AP-1-dependent transcription [13]. Furthermore, RKIP antagonizes the NF-kB signaling through its interaction with upstream kinases responsible for regulating the IkB protein while having a positive effect on the heterotrimeric G protein-coupled receptor (GPCR) and GSK signaling. RKIP also inhibits phosphorylation and activation of the transcriptional factor STAT3, suppresses the expression of NRF2-ARE containing genes, and enhances glycogen synthase kinase 3 beta (GSK3Beta)-dependent signaling [1,14,15,16,17,18,19]. Moreover, demonstrations have shown that the association of RKIP with the centrosomes and kinetochores plays a role in the regulation of the spindle checkpoint in mammalian cells [19].

Despite the well-established role of RKIP in cancer progression and aggressiveness, its role in tumor response to host immune-surveillance mechanisms and exogenous immunotherapy, as well as in the regulation of inflammatory responses, are less clear. Here, we review the literature on the pleiotropic activities and functions of RKIP and include: (i) RKIP structure, (ii) RKIP functions on cell signaling, (iii) regulation of RKIP expression, (iv) RKIP expression and tumor growth and resistance, (v) RKIP–immune system cross-talks in cancer and inflammatory diseases, (vi) bioinformatic analyses of RKIP expression levels and immune cells, and (vii) RKIP expression in cancer and inflammatory diseases [20,21].

## 2. RKIP Structure

In humans, the RKIP mRNA molecule is 1434 base pairs (bp) long and shares no significant homology with other protein families [22]. It is transcribed from a gene that contains four exons of 10 kilo base pairs (kb) located in chromosome 12q24.23 [1,6,23,24]. This mRNA encodes a protein of 187 amino acids (aa) that shares sequences with bovine and rat RKIP [1,6,7]. The human RKIP protein consists of 23kDa and 186 aa [19]. Scientists have reported that RKIP crystalizes in two asymmetric molecules of 180 and 185 residues, although information regarding its functional oligomeric and dimeric states is unknown [19,22].

RKIP’s compact structure is composed of nine-stranded beta sheets and four alpha-helices folded into a pattern that enables it to be stable and gives it its unique properties, including its conserved ligand-binding pocket [19,25,26]. The pocket is composed of 16 aa residues and accommodates several different nucleotides, including phospholipids and non-lipid organic compounds, which contribute to the column-dependent purification of the molecule [19,27,28,29]. Amino acid residues 93–134 of RKIP are responsible for the formation of the Raf-1 binding domain, where the unphosphorylated and tri-phosphorylated forms of the Raf-1 protein bind to RKIP [19,25,26].

As mentioned above, RKIP exists in multiple states, which enhances its functional switch through its flexible pocket loop [8,9]. For instance, RKIP phosphorylation at Serine153 switches it from a Raf-1 binding state to a GRK2 binding state [8,18], thus exerting different functional roles in each of the implicated signaling pathways. Unlike the RKIP-GRK2 binding state (motif), the RKIP-Raf-1 interaction motif (state) has been crystallized in several mammalian models [8,9,22,30]. Studies have further reported that RKIP has a third state that interacts with phosphorylating kinases [31].

## 3. RKIP Functions on Cell Signaling

**Figure 1:**
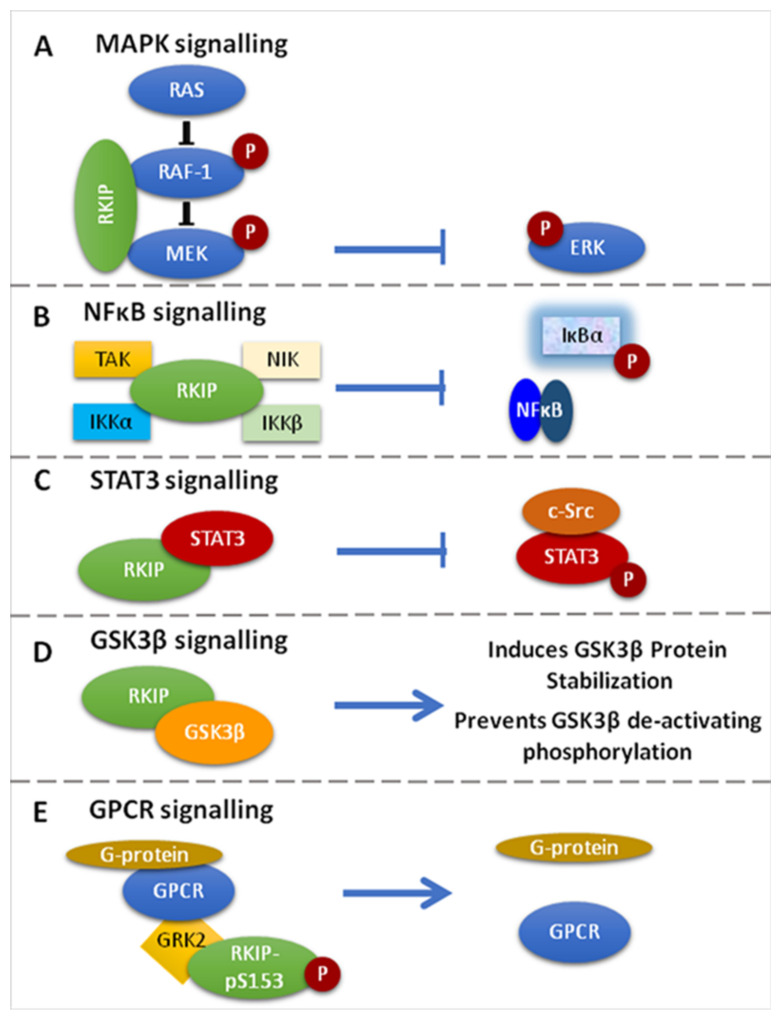
Signaling Pathways targeted by RKIP. (**A**). RKIP inhibits MAPK signaling via direct association with Raf-1 or MEK. The association prevents the phosphorylation and activation of the interacting kinases and therefore the downstream activation of ERK. (**B**). RKIP inhibits NF-κΒ signaling through interaction with the upstream kinases NIK, TAK, ΙΚΚα and IKKβ, resulting in the inhibition of IκΒα phosphorylation and the accumulation of NF-κΒ in the cytoplasm. (**C**). RKIP associates with STAT3 and inhibits its activation by preventing c-Src-mediated STAT3 phosphorylation. (**D**). RKIP interacts with GSK3β and promotes protein stabilization, while it inhibits its deactivating phosphorylation. (**E**). RKIP phosphorylated at Ser153 (RKIPpSer153) dissociates RAF-1 and binds GRK2, resulting in the de-repression of GPCR activation. Different coloured schemes in each panel represent different gene products.

Below, we briefly describe the multiple functions of RKIP in cell signaling pathways with critical involvement in cell homeostasis, survival, and disease pathogenesis [32,33] (Figure 1).

### 3.1. RKIP-Mediated Inhibition of the Raf-1/MEK/ERK Pathway

The Ras/Raf-1/MEK/ERK pathway conveys mitogenic and differentiation signals to the nucleus [13,34]. It is organized in a complex that is nucleated by Ras proteins [35] that are activated by growth factors that bind the Raf-1 kinase with high affinity. These induce Raf-1 recruitment to the cell membrane and its activation via phosphorylation. Raf-1 activation, in turn, leads to the activation of MEK, which results in the phosphorylation and activation of ERK1/2 [13,36,37]. Activated ERKs migrate to the nucleus to regulate gene expression by phosphorylating transcription factors [13,38]. Yeast studies have revealed that the JIP-1 and Ksr proteins are important scaffolding molecules used to assemble components of the MAPK pathway [13,39]. Ksr, a protein kinase, functions by binding to Raf-1, MEK, and ERK, while JIP-1 serves as a scaffolding protein for the stress-activated MAPKs/JNKs [13,40,41,42,43,44,45,46,47]. The above studies helped in the identification of RKIP as an endogenous inhibitor of the MAPK pathway via physical association with different kinases of the cascade [13,48].

RKIP can specifically inhibit the Raf-1/MEK/ERK cascade by two mechanisms: (1) by binding to the N-region of the Raf-1 kinase domain, thus inhibiting its phosphorylation and activation, and (2) by dissociating the Raf-1/MEK complex, hence inhibiting the phosphorylation and activation of MEK [1,13,14,49,50] (Figure 1A). Interestingly, it was further shown that MEK and Raf-1 bind to the same sites on RKIP, even though MEK and RKIP are associated with different domains on Raf-1. Furthermore, it was shown that Raf-1 and RKIP bind to different sites on MEK. In order for the Raf-1/MEK/ERK pathway to be suppressed by RKIP, the MEK binding sites and the Raf-1 binding sites on RKIP must be deleted. This suggests that whether RKIP binds to Raf-1 or MEK, both are ample for their inhibition [13]. Given that the MAPK signaling promotes cell migration and RKIP inhibits MAPK signaling, the restoration of RKIP expression in human hepatoma (HHC) and melanoma cells repressed tumor cell migration and motility, as well as the cellular invasive potential [51,52,53,54].

### 3.2. Inhibition of the NF-kB Pathway

Active NF-kB acts as a transcriptional regulator of several genes that play a role in immunity, inflammation, cell proliferation, cell migration, and apoptosis [51,55]. RKIP is an inhibitor of NF-κB signaling by interacting with and inhibiting upstream activating kinases, such as the transforming growth factor B-activated kinase-1 (TAK-1), the NF-κB-inducing kinase (NIK), and the IκB kinaseσ (IKKα and IKKβ) [14] (Figure 1B). The translational impact of NF-κB inhibition by RKIP in cancer conveys with cancer cell sensitization to apoptotic signals mediated by chemotherapy, or endogenous immune-related cytotoxicity or exogenous immunotherapy, inhibition of EMT and cell metastatic potential, as well as reduced cell survival [51,56]. Researchers have also proposed that since RKIP interacts with members of the NF-κB signaling pathway, it could also serve as a scaffold protein that plays a role in assembling a multicomponent protein complex [51].

### 3.3. RKIP-Mediated Inhibition of GRK2

RKIP phosphorylation at Ser153 (pSer153RKIP) increases the survival and invasion of cancerous cells by inhibiting the G-protein-coupled receptor kinase 2 (GRK-2), which negatively regulates G-protein-coupled receptor (GPCR)-mediated signaling [1]. Briefly, under normal conditions, GRK-2 binds to GPCR and blocks its activation and intracellular signaling. When RKIP is unphosphorylated, it associates with Raf-1 and suppresses MAPK signaling. RKIP is phosphorylated at Serine 153 (S153) by GPCR-induced PKC zeta [57]. In turn, this phosphorylation dissociates RKIP from Raf-1, thus de-repressing RKIP-mediated MAPK inhibition. Subsequently, the phosphorylated RKIP binds to GRK2 and desensitizes GPCRs, leading to persistent phosphorylation of Raf-1 and activation of GPCR [18,57,58,59] (Figure 1E).

High levels of pSer153 RKIP are induced by IL-6 and H. pylori infection in colon and gastric cancers, respectively, and are associated with a poor prognosis in stage II colon cancer patients and little to no response to therapy for patients with multiple myeloma [57,60,61]. In gastric cancer models, H. pylori-mediated phosphorylation of RKIP at S153 promotes its nuclear accumulation in cancer cells, while it targets unphosphorylated RKIP for proteasome degradation [62,63]. Unlike the unphosphorylated RKIP form, the pRKIP acts by competitively inhibiting survival signals and promoting apoptosis in cancer cells [64,65,66].

### 3.4. RKIP-Mediated Inhibition of STAT3 Activation

STAT3 is a member of the signal transducer and activator of transcription (STAT) family, which is found in the cytoplasm, and upon being activated by phosphorylation, it translocates in the nucleus acting as a transcription factor for genes involved in the process of apoptosis, cell growth, survival, and differentiation [1,67]. Although the exact mechanism is unknown, studies in colon cancer models have shown that the overexpression of RKIP inhibits IL-6-, Jak-, or Src kinase-mediated phosphorylation of STAT3 known to be necessary for its activation [1,17] (Figure 1C).

### 3.5. Regulation of GSK3β Signaling

RKIP has been reported to regulate glycogen synthase kinase 3 (GSK3β) levels by (1) direct binding to the GSK3β protein that results in better protein stabilization and (2) by preventing GSK3β inhibitory phosphorylation [51] (Figure 1D). Depletion of RKIP induces high levels of oxidative stress response that leads to p38 MAPK activation, which ultimately inhibits GSK3β by phosphorylating its T390 residue, an inhibitory residue [51,68,69]. Furthermore, when RKIP is depleted, downstream GSK3β targets are activated, leading to cyclin D1 stabilization, which is what induces cell cycle progression and expression of β-catenin, SNAIL and SLUG. These three are also responsible for promoting the invasion and EMT [51,69]. In addition, in HEK-293 cells where RKIP was depleted, cell migration was favored by inducing p38-mediated phosphorylation of GSK3β, whereas its degradation stabilized cell migration regulatory molecules, such as β-catenin [51,69].

### 3.6. Regulation of the Spindle Checkpoint by RKIP

RKIP also regulates the spindle checkpoint and thus plays a role in the control of the cell cycle and the stability of the genome by associating with the centrosomes and kinetochores in mammalian cells [51,70,71]. RKIP-depleted cells rapidly move to the anaphase displaying a defective spindle checkpoint. Its depletion led to the reduced localization and kinase activity of Aurora B, a kinase that helps with chromosomal alignment as well as spindle checkpoints and cell division [51,70,72]. Furthermore, RKIP deficient cells tend to display decreased localization of Aurora B to kinetochores, which leads to inhibition of the activity of its kinase because of the hyperactivation of the MAPK pathway. This process sometimes leads to chromosomal abnormalities [51,70]. Using comparative genomic hybridization and allelotyping, researchers found that colorectal tumors lacking or weakly expressing RKIP display chromosomal losses and are genomically unstable, unlike cancers expressing it [51,73]. In addition, cells with depleted RKIP exert a shorter transition time from their nuclear envelope breakdown to anaphase, which, together with Aurora B and G2/M downregulation, gives cells a highly proliferative quality due to a faster rate of completion of the cell cycle phases [51,69]. This data further proposes that RKIP does in fact have an influence on cell proliferation and its overexpression reduces cell growth compared to RKIP depleted cells [51]. The depletion of RKIP indirectly influences the acceleration of cellular proliferation and growth by means of modulating the expression of genes that are involved in DNA replication, transition through G1/S phase, G2/M checkpoints, and genomic stability [51,69].

### 3.7. Clinical Significance of pRKIP in Various Cancers

#### 3.7.1. pRKIP Expression Correlates with Good Prognosis

Huerta-Yepez et al., (2011) studied the expression of pRKIP and RKIP in NSCLC patients in order to determine the ability of these proteins to predict prognosis [65]. Using Western blot analysis, researchers studied three different lung cancer cell lines to see the levels of pRKIP and RKIP, which they found to be different for all the 3 cell types [65]. Although RKIP expression was constant in nonmetastatic and invasive and metastatic cases, pRKIP expression was decreased in invasive cancers compared to non-malignant cancers [65]. Furthermore, the significance of RKIP and pRKIP levels as a predictor of prognosis was tested via the Cox model analysis and was first found to have no significant effect on prognosis. A second trial was run using dichotomized RKIP and pRKIP expression and it was found that a higher expression of pRKIP did in fact lead to a greater survival of patients with NSCLC compared to those with lower expression of pRKIP [65]. More specifically, researchers found that patients that had higher levels of pRKIP were mostly older than 65 and that in patients younger than 65, high levels of pRKIP were not necessarily an indicator of survival. In addition, high levels of pRKIP served as a marker of good prognosis in early-stage NSCLC but not in the later stages of the disease [65]. 

Studies have also shown that a loss or reduction in phosphorylated RKIP expression in patients with breast cancer is interrelated with poor disease-free survival [64,65]. A study done by Al-Mulla et al., determined that when there was a reduction in RKIP expression or a loss of expression, patients with breast cancer often times were prone to larger sized tumors with substantial necrosis and a higher tumor grade [51]. Furthermore, by looking at data from 115 women with breast cancer that were previously studied, they found that in women with higher RKIP gene expression, the cancer cells tended to be non-metastatic while those with lower gene expression had cells that became metastatic [51]. Patients with low RKIP expression also exhibited significantly lower disease-free survival compared to patients with tumors that had high RKIP mRNA levels. These data suggest that higher levels of pRKIP, which lead to higher expression of RKIP, correlate with a good prognosis. However, limited and contradictory data are available when it comes to the clinical implications of pRKIP in tumors. A study done by Li et al., (2016) was the first to show that pSer153RKIP is a favorable prognostic factor for patients with nasopharyngeal carcinoma (NPC) who received radiation [64], while in patients with early-stage lung cancer, normal expression of phosphorylated RKIP was an indicator of more favorable survival [51,64]. On the other hand, RKIP is phosphorylated in other tumors, including multiple myeloma and stage II colon cancer, positively contributing to cell survival and drug resistance, which is mainly done through the transcription of downstream anti-apoptotic genes [60,64,66].

#### 3.7.2. pRKIP Expression Correlates with Poor Prognosis

Cross-Knorr et al., (2013) performed a study demonstrating the effects of IL-6-mediated activation of STAT3 on the phosphorylation of RKIP [60]. They performed this study on colon cancer cells of humans. Increased IL-6 stimulation is common in various cell lines and tumors and is linked to cancer metastasis and cancer cell survival as a result of STAT3 phosphorylation [60,74,75,76,77,78,79,80]. Specifically, in colon cancer there is often an increase in the soluble receptor of IL-6 rather than the membrane receptor, leading to an increase in the activation of STAT and hence the activation of pro-survival proteins [60,81,82]. The activation of STAT3 by IL-6 in colon cancer was studied by examining HCT116 cells after IL-6 treatment, and specifically looking at STAT3 and pRKIP levels [60]. Furthermore, after questioning the effects of STAT3 overexpression on transcription and pRKIP, Cross-Knorr et al., performed Western blot analyses and found that when transfected with STAT3, the levels of expression of phosphorylated RKIP did in fact increase [60]. This increase in expression has led to a poor prognosis in patients with stage II colon cancer. A Kaplan–Meier analysis was done on a group of patients and indicated that elevated pRKIP was correlated with a decrease in the survival of patients and that patients with lower levels of pRKIP had lower levels of lymphovascular invasion than those with higher levels of pRKIP [60].

This study had different results to the study done by Huerta-Yepez et al., who found that in patients with non-small cell lung cancer, those who have normal pRKIP levels are more likely to have a better prognosis [83]. It is hypothesized that these two studies may have had different results because the experiments were run using different types of tissues or because when pRKIP is phosphorylated, a number of distinct pathways can be activated, which would result in worse or better prognosis in patients depending on which pathways were active. In conclusion, patients with stage II colon cancer exhibiting high levels of pRKIP had poor prognosis and shorter recurrence-free survival [60].

## 4. Regulation of RKIP Expression 

**Figure 2:**
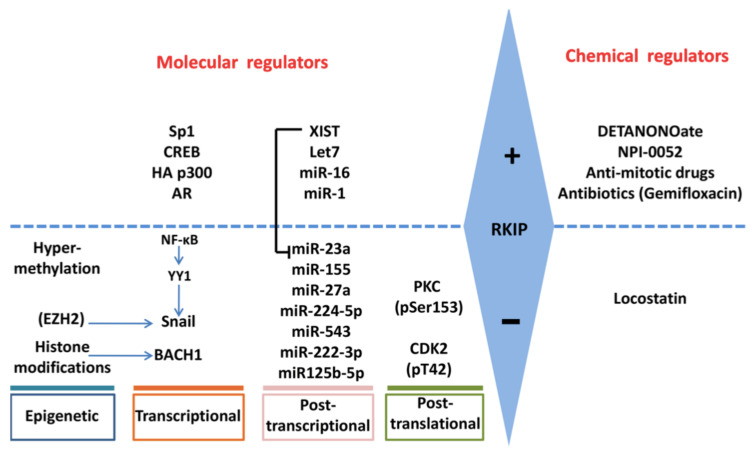
Regulation of RKIP expression. RKIP expression is regulated positively or negatively by multiple gene products and processes at epigenetic, transcriptional, post-transcriptional and post-translational levels. The involvement of each molecular regulator is cancer-type dependent. RKIP levels may also be modified indirectly by chemical molecules that may target the direct RKIP regulators.

Below, we describe the different ways that RKIP is regulated and the different regulators responsible for its regulation (Figure 2).

### 4.1. SNAIL

The most well-known transcriptional regulator of RKIP is the epithelial-to-mesenchymal transition (EMT) protein SNAIL. Researchers have demonstrated a negative correlation between SNAIL and RKIP expression in prostate cancer and identified SNAIL as a direct transcriptional repressor. It was proven that SNAIL expression can be induced by NFκB and the transcription factor Yin Yang 1 (YY1), leading to RKIP downregulation [62,84,85]. Furthermore, in melanoma cell lines, a PDZ-domain (the postsynaptic density protein (PSD-95), melanoma differentiation associated gene 9 (MDA-9), discs-large tumor suppressor (Dlg), and tight junction protein-1 (*Z*O-1)) contains a scaffold protein that can silence RKIP transcription by activating SNAIL expression through ERK1/2 and NFκB signaling due to its role in promoting melanoma progression [54,62].

As for the chemical induction of RKIP, the organic molecule nitric oxide was found to be able to induce RKIP expression [62,86,87]. A nitric oxide donor, DETANONOate, inhibits NFκB signaling and lifts SNAIL-mediated transcriptional suppression on the RKIP promoter [62,86]. In some cases, chemotherapy, immunotherapy, and radiotherapy can also induce RKIP expression.

Snail has been shown to strongly repress E-cadherin transcription. Due to the coexpression between RKIP and E-cadherin, the hypothesis was that Snail may also act similarly towards RKIP [84,88,89]. Suppression of RKIP expression is inversely correlated with the expression levels of Snail; thus, when Snail expression was knocked down by specific siRNA, RKIP expression increased, suggesting that Snail is a repressor of RKIP [84]. Snail represses RKIP by binding to the E-box cis-elements in the RKIP promoter and recruiting mSin3A histone deacetylases and transcriptional repressor complexes [84,90,91]. When Snail is present, the enhancer of Zeste homolog 2 (EZH2) inhibits RKIP expression at the transcriptional level.

### 4.2. BACH1

BACH1 expression is negatively correlated with the expression of RKIP in breast cancer, suggesting that it may be its key negative regulator [62]. Similar to Snail, BACH1 positively correlates with the expression of EMT-associated genes, implying that it plays a role in EMT [62]. In addition, both molecules negatively regulate their own promoters, reducing their expression in a negative feedback loop manner. In addition, both are downstream targets of RKIP but also its negative regulators [62,92].

### 4.3. SP1, CREB, p300, AR

The region spanning from −56 to +261 nucleotides in the promoter region of RKIP is needed for its full activity. In this region, cis-acting elements that respond to Sp1, CREB, and histone acetylase p300 are needed for the maintenance of its promoter activity [57,93]. For example, studies have shown that knocking down CREB, p300, or Sp1 or the mutation or deletion of these elements limited RKIP promoter activity, meaning that they positively regulate RKIP transcription [57,93]. Finally, in prostate cells, the binding of the androgen receptor to an androgen responsive element in the RKIP promoter has a positive regulatory effect on the regulation of its transcription [57,94].

### 4.4. c-MET

The transcription factor C-MET activates β-catenin and acts as a growth factor that promotes cell migration, whereas PAK1 is a protein kinase involved in cell motility. Both molecules were found to be upregulated in RKIP-silenced HEK-293 cells [51,95,96], supporting the idea that RKIP loss influences the cell proteome and transcriptome to favor migration.

### 4.5. MMPs

It has been hypothesized that RKIP’s suppressive role in metastasis and cell migration is due to its ability to downregulate specific matrix metalloproteinases (MMPs) expression, such as MMP-1 and MMP-2 [51,97]. High expression of MMP-1 and MMP-2 along with a high invasive cellular property as a result of RKIP silencing led to the conclusion that RKIP controls the invasion of cancer cells by negatively regulating NF-kB, which in turn controls the expression of MMP and, therefore, cellular invasion [51,97].

### 4.6. EZH2

It is known that the downregulation of EZH2 inhibits cancer cell growth, proliferation, and invasion [98,99]. A decreased EZH2 expression can also increase the expression of RKIP in cancer cells [98]. An experiment was done to test whether decreasing cellular proliferation, growth, or invasion via silencing EZH2 could be reversed if RKIP expression was silenced. The inhibition of RKIP had no effect on cellular growth or proliferation, but it did effectively reverse the decrease in invasiveness that was caused by the loss of EZH2 [98], suggesting that the EZH2-mediated inhibition of RKIP is part of the molecular mechanism by which EZH2 promotes cellular invasion and metastasis in prostate and breast malignancies.

### 4.7. Methylation

The quantification of RKIP transcript levels in different cancer cell lines suggested that its downregulation is due to changes in the stability of its mRNA or the initiation of transcription [84]. Another study was performed to determine whether RKIP is repressed by methylation in metastatic prostate cell lines by examining the effects of trichostatin A (TSA, a histone deacetylase (HDAC) inhibitor) on RKIP expression. The authors reported that TSA significantly increased RKIP expression in the human prostate carcinoma cell line, DU145, but treatment with 3 microM 5-Aza-2dC, a demethylation agent, had no effect on RKIP expression [84,100]. The cellular treatment with 5-Aza-2dC showed that hyper-methylation is not the cause of RKIP downregulation. In addition, the fact that RKIP expression can be induced by TSA in cancer cells implies that it may be actively repressed in them [84]. Several studies on the promoter methylation have shown that the methylation status of the RKIP promoter is correlated with low RKIP expression levels in advanced stages of several tumors, including gastric adenocarcinomas, esophageal squamous cell carcinomas, and colorectal and breast cancers [62,73,101,102,103,104,105,106]. In esophageal and gastric cancers, the RKIP promoter was significantly hypermethylated in poorly differentiated tumors and lymph node metastases and this hypermethylation was associated with worse overall survival [62,102,104]. Furthermore, in the prostate cancer cell line DU145, TSA caused an increase in RKIP levels [62,84], which raises the question of whether histone deacetylation plays a role in RKIP silencing or not. One study showed that treatment of the triple-negative breast cancer (TNBC) cell line SUM159 with histone deacetylase inhibitors induced RKIP expression [107], but this was not verified in a different study [92]. Further research on this noted that BACH1 was induced by treatment with an HDAC inhibitor [62,92]. The authors also confirmed that EZH2 interacts with BACH1 in the TNBC cells to inhibit RKIP transcription, which can also be seen in breast and prostate cancer cells by interaction with Snail [62,98].

### 4.8. miRNAs

As seen in previous studies, RKIP’s expression is inversely correlated with miR-23a in prostate cancer and other malignancies [108,109,110]. To confirm the involvement of miR-23a in the regulation of RKIP in prostate cancer, Du et al., (2017b) computationally predicted that miR-23a binds directly to the 3′UTR of RKIP [108]. This prediction was verified using luciferase reporter assays. Co-transfection with luciferase reporter plasmids and miR-23a mimics showed that the luciferase activity of RKIP was significantly inhibited [108]. The knockdown of miR-23a also led to a significant increase in the expression of RKIP, whereas over-expression of miR-23a caused a significant reduction in RKIP protein expression [108]. Overall, the above results indicate that miR-23a negatively regulates RKIP in prostate cancer cells. Furthermore, RKIP gene expression can be inhibited by certain microRNAs [111], but the overexpression of let-7, miR-1, and miR-16 can enhance RKIP protein translation. On the other hand, the overexpression of miR-155 can destabilize RKIP and reduces its expression [90,91,112,113].

RKIP is reduced in metastasis and has been especially studied in breast cancer cells [114,115]. Several miRNAs, including miR-224, miR-27a, miR-23a, and miR-543 have been shown to target and inhibit the RKIP transcript [102,108,110,114,116,117]. Of these, only miR-224 seems to inhibit RKIP expression in breast cancer [111,114]. After looking at the relationship of RKIP expression and 2238 miRNAs, researchers classified three miRNAs as putative RKIP expression-regulating molecules: miR-224-5p, miR-222-3p and miR-125b-5p [110,111,114]. These were selected based on their correlative expression with RKIP in breast cancer cell lines [84,114]. To establish a regulating effect of the miRNAs on RKIP, three conditions need to be applied. They are: (i) negatively correlated with its expression levels, (ii) physically associated with the RKIP mRNA molecule, and (iii) the existence of miRNA recognition elements (MREs) for the identified miRNA in the 3′UTR and amino acid coding sequence of the RKIP gene is required [110,114]. Experiments showed that miR-224-5p functions as an RKIP repressor in breast cancer cells [111,114,118].

The miR-224 can negatively regulate RKIP, contributing to increased cell proliferation and invasion in gastric and breast tumors and in hepatocellular carcinoma [57,117,119,120]. RKIP can also be targeted by miR-27a, the upregulation of which contributes to chemoresistance in lung adenocarcinoma [57,117].

### 4.9. PKC

The protein kinase C (PKC) phosphorylates RKIP, activating its downstream signaling pathways [121]. PKC signal transduction participates in the process of T cell apoptosis and proliferation. It is the key enzyme during the cells inner signal transduction, which can regulate the expression of IL-2, IL-6, GM-CSF, and IL-1B by means of activating the backward position of NF-kB and AP-1 [122]. PKC also plays an important role in the immune inflammation reaction, cell apoptosis and proliferation, and self-immunity disease. PKC also plays a role in the mechanisms by which asthma works [122,123]. PKC phosphorylates RKIP at serine 153, dissociating it from Raf-1 [124].

### 4.10. XIST

Du et al., (2017b) investigated whether x-inactive specific transcript (XIST) acts as a competing endogenous RNA (ceRNA) in RKIP regulation, using immunoblotting assays to determine its expression post transfection with XIST (or si-XIST) and miR-23a mimics (or inhibitors, respectively) [108]. The results revealed that XIST overexpression substantially promoted RKIP expression, whereas miR-23a repudiated it [108]. While this was the case for overexpression of XIST, researchers found that the knockdown of XIST led to a reduction in RKIP that could essentially be returned by the inhibition of miR-23a [108]. All in all, these findings suggest that lncRNA XIST regulates RKIP expression in a ceRNA manner and miR-23a plays a main role in XIST-mediated regulatory pathways.

XIST has been known to be responsible for suppressing cellular proliferation and metastasis in prostate cancer cell lines and for negatively regulating miR-23a expression [108]. While investigating the correlation between XIST and miR-23a, miR-23a was identified as a direct target of XIST and its over-expression could overturn the XIST-induced RKIP up-regulation [108]. This suggests that XIST positively regulates RKIP expression through miR-23a binding.

## 5. Effects of RKIP Expression Levels in Various Cancers

In humans and other mammals, NF-kB binds to the promoter and enhancer sequences in various cells. It plays a known role in immune response, inflammatory response, and cell growth regulation through its ability to regulate adhesion molecules, immune receptors, and chemotactic and growth factors [125,126]. This study also showed that NF-kB enhances the host defense function of macrophages and neutrophil granulocytes and has a function in antigen presentation in dendritic cells and T cell activation [125]. Therefore, the degradation of NF-kB or the knockdown of NF-kB-dependent genes can lead to immune dysfunction in the host [127], and the overexpression of RKIP can lead to a decreased level of NF-kB-mediated responses [125]. In healthy patients, the immune system maintains a stable state with T-lymphocytes and Treg cells. It has been useful to understand that the gene products of mice known as cluster of differentiation (CD) can recognize the antigenic determinant on the surface of these T lymphocytes [125]. CD3 is the surface marker for mature T cells, CD4 is the marker of T helper cells, and CD8 is the marker of cytotoxic T cells. A decline in CD3 cells and an abnormal CD4/CD8 ratio indicate impaired cellular immunity [125]. Wei et al., (2015) found that CD3^+^ and CD4^+^ cell counts and CD4^+^/CD8^+^ ratios in gastric cardia adenocarcinoma cases were lower than in the control [125]. They also noted that CD8+ and Treg cell counts of gastric cardia adenocarcinoma were higher than in the control. These variations were all related to the levels of RKIP expression. Overall, it was hypothesized that the downregulation or deletion of RKIP has an effect on the NF-kB-mediated positive feedback mechanism and leads to the activation of upstream regulators [125,128]. The deletion of these genes, in turn, causes the inhibition of NF-kB activity and a steep decline in cellular immunity. In conclusion, RKIP expression is able to closely associate itself with metastasis and progression of gastric cardia adenocarcinoma tumors because of the inhibition of NF-kB activity and cellular immunity, which enables tumor cells to evade immune surveillance.

RKIP plays a major role in the survival of cancer patients. Two hyperactivated pathways in cancers are the MAPK/ERK and NF-kB pathways, both being regulated negatively by RKIP. The ERK1/2 are well-known as downstream effectors of the MAPK pathway that phosphorylate and activate several transcription factors, including CREB, c-Myc, and NF-kB, all of which play a role in regulating cell proliferation, differentiation, and survival [129,130]. In addition to suppressing the activating phosphorylation of ERK, RKIP also inhibits cell proliferation and promotes cell death by ablating MAPK signaling [48,129,130,131,132,133,134]. More specifically, the loss of RKIP function correlates with the inactivation of aurora B kinase through the hyperactivation of the Raf/MEK/ERK signaling cascade [70,129]. Interestingly, bypassing this checkpoint can result in chromosomal abnormalities, and the extent of genomic instability is measured by chromosomal losses, which are inversely proportional to the expression levels of RKIP in colorectal cancer [73,129].

NF-kB is a pro-survival transcription factor that exerts its activity through effector proteins and RKIP has a direct influence on its activation by inhibiting upstream kinases including NIK, TAK1, and IKK [14,129]. The inactivation of NF-kB by RKIP is important because it is most notably a participant in the pro-survival and anti-apoptotic pathway called the NF-kB/Snail/YY1/RKIP/PTEN dysregulated loop [129,135]. The induction of RKIP overexpression is able to interrupt the anti-apoptotic natural properties of the loop [136,137], while Snail and YY1, NF-kB-regulated factors, promote anti-apoptotic and pro-metastatic genes and serve as negative regulators of RKIP expression [129,138]. Another important molecule that plays a role in the regulation of cell cycle and survival is the transcription factor STAT3. RKIP blocks IL-6-, Janus kinase 1/2 (JAK1/2)-, and Raf-mediated activation of STAT3 by c-Src and c-Src autophosphorylation [129,139]. Overexpression of RKIP, in turn, enhances apoptosis by suppressing STAT3 targets. From a clinical standpoint, high levels of nuclear pRKIP and STAT3 are correlated with poor prognosis in stage II colon cancer patients, suggesting that RKIP regulates STAT3-mediated cell survival [60,129].

Ying Yang 1 (YY1) promotes therapeutic resistance in solid and hematological malignancies [12,134,135,140,141,142]. YY1 is regulated by the inhibition of NF-kB by RKIP since it functions downstream of NF-kB. This, in turn, eliminates the effect that it has on the resistance of cancer cells [12,57,135,143]. YY1 inhibition induced by RKIP contributes to Snail suppression since YY1 is known to directly act as a Snail transcription activator [12,57,136,144]. Therefore, YY1 might function as a link between NF-kB and Snail activation, which affects the activity of downstream cell death pathways [12,85,135]. Because of this, RKIP’s ability towards the NF-kB/YY1/Snail circuit is thought to be the underlying mechanisms of RKIP-mediated inhibition of tumor chemoresistance and immune-resistance [12,57,135].

Below, we give a few examples of different cancers in which RKIP plays a role (Table 1).

### 5.1. Adenocarcinomas

RKIP is widely distributed across different human tissues [12,23,53,115,125]. In patients with adenocarcinoma, the deletion or downregulation of the *RKIP* gene was predicted to result in a poor prognosis [125]. Scientists found that in gastric cardia adenocarcinoma, the expression of RKIP was substantially lower in precancerous tissues in patients with lymph node metastasis, which further implies that RKIP plays a role in the progression, metastasis, and invasion of gastric cardia adenocarcinoma [125,145].

### 5.2. Colon Cancer

A further study showed that the overexpression of Snail correlates with high expression of cancer stem cell (CSC) markers and increased chemoresistance in colon cancer cell lines [12]. When looking at the effects of Snail silencing and RKIP upregulation, it was seen that the Snail/RKIP loop is an essential component of CSC existence within the tumor, associated with regulation and tumor chemoresistance [12,146]. The reduction of RKIP led to amplified radio-resistance of non-small-cell lung cancer (NSCLC), which generally accelerates the expression of CSC markers and sustains properties of CSC via the expression of Snail [12,147,148]. This demonstrates that RKIP has a negative effect on radio-resistance regulation and chemoresistance because it affects the number and function of CSCs in the tumor.

### 5.3. Prostate Cancer

Initially, RKIP was found to play a role in cancer through a gene array analysis that was performed to determine the genes that regulate metastasis. Researchers discovered that the prostate cancer (PCa)-associated LNCaP cell line has a low metastatic rate and therefore expresses higher RKIP levels than the cells of the derivative cell line C4-2B, which contained a high metastatic rate [1,23]. When C4-2B PCa cells were transfected with RKIP, its expression was restored and led to a reduction in spontaneous lung metastasis, but not primary tumor growth, proving that RKIP functions as a metastasis suppressor gene [1,56,149]. In addition, the restoration of RKIP expression was shown to inhibit breast cancer metastasis in murine models [91,97]. The downregulation of RKIP induced a high viability and migration of cells but did not have an effect on angiogenesis and cellular proliferation [1,150]. RKIP acts as an inhibitor at the molecular level and its loss promotes metastasis through the inhibition of angiogenesis, local invasion, colonization, and intravasation [1,23,91]. It is possible that the inhibitory protein does this by modulating the extracellular matrix [1,151]. RKIP also acts as a phosphorylation target for pathways that involve MAP kinase (MAPK) and the beta-adrenergic receptor (B-AR) and regulates these pathways by binding as well as inhibiting Raf and G protein-coupled receptor kinase 2 (GRK2) [8,18].

It has been found that RKIP expression is downregulated in several tumors, including those of highly metastatic prostate, colon, and breast cancer, hepatocellular carcinoma, and skin melanomas [12,23,52,53,56,84,115,149,152,153]. Furthermore, the restoration of its expression inhibits prostate cancer metastasis [23,84,154]. In order to study the regulation of RKIP, researchers examined its expression in cancer cell lines with different metastatic capacity. They observed that in highly invasive, metastatic cancers, such as breast and prostate cancers, its expression is repressed, while in noninvasive cell lines RKIP expression is high [84]. Researchers also found that RKIP is correlated with the intracellular adhesion protein E-cadherin (E-cad), which is regulated by the Snail and Slug transcription factors [84,155].

Prostate cancer accounts for 15% of cancers diagnosed in males and for 13% of cancer-related deaths [108,156]. In early state prostate cancer, tumor growth is generally attributed to the presence of androgens in the body. Androgen deprivation therapy (ADT) is the main form of treatment used for androgen-dependent prostate cancer, but a large majority of the tumors tend to continue to grow after remission that lasts about 18–24 months and continue to do so in an androgen-independent manner [108,157,158]. The long non-coding RNAs (lncRNAs) are a relatively new form of non-encoding RNA transcripts (>200 nucleotides) [108,159] that participate in cellular development and differentiation, as well as in tumorigenesis. They can also regulate gene expression in multiple ways, thus affecting several processes, including chromatin structure, nuclear transport, cutting and splicing, transcriptional modification, epigenetic control, and RNA decay [108,160,161,162,163,164,165]. LncRNAs have also been observed in many cancers and studies indicate that they can act as tumor suppressors, oncogenes, or even both [108,166]. For instance, Zeng et al., (2017) reported that lncRNA AF113014 acts as a tumor suppressor in hepatocellular carcinoma cells by promoting Egr2 expression [167], and Li et al., (2017) showed that the lncRNA n340790 enhances cellular proliferation in thyroid cancer by means of targeting miR-1254 [168]. In a study by Du et al. (2017), it was confirmed that the downregulation of XIST in prostate cancer is a common molecular change [116]. The expression of XIST is negatively correlated with metastasis and its low expression is associated with poor prognosis in prostate cancer patients. Furthermore, the overexpression of XIST has the potential to inhibit proliferation, migration, and invasion in prostate cancer cells [108].

Scientists have found evidence that the expression of lncRNAs is strongly associated with the development of cancer. Studies have shown that the lncRNA XIST regulates different cancers but in prostate cancer its underlying mechanism is still unclear [108]. A downregulation of XIST in prostate cancer specimens and cell lines that leads to a low expression of XIST has been correlated with advanced tumor stage in patients with prostate cancer and a poor prognosis in those patients [108].

### 5.4. Pancreatic Cancer

A study done in 2012 aimed at assessing whether (-)-epigallocatechin 3-gallate (EGCG) regulates the expression of RKIP and invasive metastatic activity in AsPC-1 pancreatic adenocarcinoma cells via epigenetic modifications. Results showed that RKIP expression differed in human pancreatic cancer cell lines. Baseline levels of RKIP were investigated in the cell lines MIA PaCa-2, PANC-1, AsPC-1, and BxPC-3. The AsPC-1 cell line was specifically selected to find out whether RKIP expression was in fact induced by EGCG treatment since it had the lowest level of RKIP of all the chosen cell lines [169]. An MTT assay was used to examine the effect of EGCG on AsPC-1 viability and it was revealed that no toxicity was present up to 10 μM EGCG, which is less than the usual EGCG treatment which is 15 μM [169]. Treatment with 10 μM of EGCG for 24 h led to an increase in RKIP expression in AsPC-1 cells compared to control cells, and in order to confirm if this RKIP regulation was due to histone modification, researchers performed the same experiments along with cellular treatment with TSA [169]. They found that in the presence of TSA, RKIP expression was induced and that the effects were synergistic to the effects of EGCG, allowing them to conclude that in AsPC-1 cells treated with EGCG, RKIP induction is partly due to HDAC modifications [169]. Furthermore, to explain the mechanism in which EGCG inhibits invasion and metastasis, researchers studied the expression of metastasis-related genes, such as *MMP-2* and *-9*, *Snail* and *E-cadherin*. Results revealed that compared to the mRNA and protein levels in the control cells, the levels in MMP-2, MMP-9, pERK, and Snail were downregulated in the EGCG-treated AsPC-1 cells [169]. E-cadherin was notably increased by EGCG treatment. Since RKIP is responsible for regulating NF-kB activation via the MEK/ERK signaling pathway, the next step was to investigate whether treatment with EGCG inhibits the ERK phosphorylation [169,170]. The results of this experiment showed that treatment of cells with EGCG suppressed ERK phosphorylation and increased RKIP expression. Essentially, the results proved that when RKIP expression is induced by EGCG, it in turn inhibits the phosphorylation of ERK and NF-κB activation while also decreasing Snail expression, which means that EGCG acts as an HDAC inhibitor and can prevent the invasive metastasis of human PC cells. In conclusion, the results of this study showed that EGCG induced RKIP upregulation through the inhibition of HDAC activity, which in turn increases histone H3 expression and inhibits Snail expression, NF-κB nuclear translocation, MMP-2 and MMP-9 activity, and Matrigel invasion in AsPC-1 cells [169]. The results also infer that EGCG regulates RKIP/ERK/NF-κB and/or RKIP/NF-κB/Snail, as well as inhibiting invasive metastasis in the AsPC-1 human pancreatic adenocarcinoma cell line.

### 5.5. Gliomas

Gliomas are tumors that are aggressive and there are no cures currently. Prior reports have indicated that there was a good correlation between the low expression of RKIP and a higher tumor grade [171,172]. The expression of RKIP in gliomas and its clinical significance in metastasis has been reported [150]. The findings by Martinho et al., were different from the report by Maresch et al., of the correlation between the loss of RKIP and high malignant grade [150,171]. However, the association between the loss of RKIP expression and the poor prognosis of high-grade gliomas reported by Maresch et al., was consistent with the findings of Martinho et al. [150,171]. In gliomas, RKIP expression did not affect cell proliferation, and downregulation of RKIP enhanced cell migration but did not affect tumor angiogenesis [150] in contrast to other murine cancers such as prostate [23] and breast cancers [173]. Overall, these findings suggested that the loss of RKIP expression correlates with a poor clinical outcome in glioma patients. 

### 5.6. Renal Cell Carcinoma

Clear cell renal cell carcinoma (ccRCC) is a cancer in which there are no reliable biomarkers for either its diagnosis or its prognosis. Papale et al., reported that urine from patients with ccRCC has high levels of RKIP and phospho-RKIP that predicted cell survival and progression-free survival [174]. Down-regulation of RKIP expression has been implicated in the development and progression of renal cell carcinoma (RCC). Recently, a study of 310 RCC cases has suggested that RKIP was a significant prognostic marker because of its close correlation with progression and metastasis of RCC. Furthermore, reduced RKIP expression was related to later disease stage, larger tumor size, sarcomatoid subtype, and poor overall survival. These authors also reported that the genetic polymorphisms in RKIP might be associated with the susceptibility and progression of RCC [175].

### 5.7. Gastric Cancer

RKIP expression in intestinal-type gastric cancer was reported to be significantly lower and the authors proposed that RKIP is an independent prognostic factor for intestinal gastric cancer [17]. Furthermore, immunohistochemical analysis showed that the RKIP expression level was the highest in nonneoplastic gastric tissue, low in primary gastric cancer tissue, and the lowest in metastatic gastric cancer tissue, suggesting that RKIP may play a role in the tumorigenesis and metastasis of gastric cancer [176]. Additional studies revealed that RKIP protein expression was negatively correlated with the depth of invasion, TNM stage, and lymph node metastasis. Further clinical and pathological analyses revealed that RKIP protein expression was negatively correlated with the depth of invasion, TNM stage, and lymph node metastasis [132,176,177].

### 5.8. Lung Cancer

Lung cancer is the most common and fatal for both male and females. Lung cancer is usually divided into two main categories: small cell lung cancer (SCLC) and non-small cell lung cancer (NSCLC). The latter is further divided into three major types, squamous cell carcinoma (SCC), adenocarcinoma (AC), and large cell carcinoma [178]. Lung AC is the most common type of lung cancer and has a poor survival rate. Studies on the RKIP expression and its clinical significance in lung cancer are not very conclusive. There is one study that assessed the expression levels of the inactive form of RKIP with phosphorylation at serine 153 (pRKIP) [65]. In agreement with this, studies in melanoma and breast cancer have also shown that low levels of pRKIP could predict poor survival in comparison with relatively higher expression [19,179]. Studies by our group analyzed RKIP mRNA expression across 37 different cancer types and, using data from The Cancer Genome Atlas (TCGA) platform, showed that RKIP is downregulated in lung cancer compared to normal lung tissues, with lung adenocarcinoma being among the eight tumor types with the lowest RKIP expression levels [12]. 

### 5.9. Leukemia

RKIP plays a major role in physiologic hematopoiesis and myeloid malignancies. In physiologic hematopoiesis, a decrease in RKIP expression in the HSPC pool increases the myelomonocytic lineage commitment of these cells. RKIP loss has been described in acute myeloid leukemia (AML) and a series of other myeloid neoplasia (MNs), and a functional involvement in myeloid leukemogenesis has been proven. These same authors have shown that RKIP inhibits proliferation and transformation of myeloid cells and decreases the transformation that is induced by mutant RAS. Both in vitro and in vivo experiments demonstrated that RKIP is an essential player within the development of these liquid tumors. They postulated that RKIP expression is of prognostic relevance and is a target for enhancing therapeutic strategies in AML [180].

### 5.10. Multiple Myeloma

Multiple myeloma (MM) is a clonal plasma-cell neoplastic disorder arising from an indolent premalignant disease known as monoclonal gammopathy of undetermined significance (MGUS). All tumors examined have exhibited low levels of RKIP; in contrast, RKIP is overexpressed primarily in its inactive phosphorylated form in MM cell lines and patient-derived tumor tissues. RKIP and the inactivated p-Ser153 form of RKIP are overexpressed in multiple myeloma cell lines and patients’ tissues compared to other tumors, healthy B cells, and healthy bone marrow. Specifically, about half of the RKIP positive cells in MM are in the phosphorylated form. The high RKIP expression in MM is positively correlated with a more aggressive diagnosis, usually resulting in a worse prognosis [90]. 

### 5.11. Other Cancers

RKIP has been identified as an important protein in various cancer types, several of which have been described above. In the majority of different types of cancers, RKIP exhibits low expression levels and RKIP is often absent in metastasis. RKIP loss has been suggested to result from the hypermethylation of its promoter. RKIP mRNA expression across 37 different cancer types was measured using data from The Cancer Genome Atlas (TCGA) platform, corroborating its downregulation in the majority of them compared to the normal tissues. This analysis showed that RKIP exhibits its highest levels in adrenocortical carcinoma (ACC), liver hepatocellular carcinoma (LIHC), and thyroid carcinoma (THCA), and its lowest expression was detected in acute myeloid leukemia (LAML), esophageal carcinoma (ESCA), and stomach and esophageal carcinomas (STES) [12].

## 6. RKIP–Immune System Cross-Talks 

**Figure 3:**
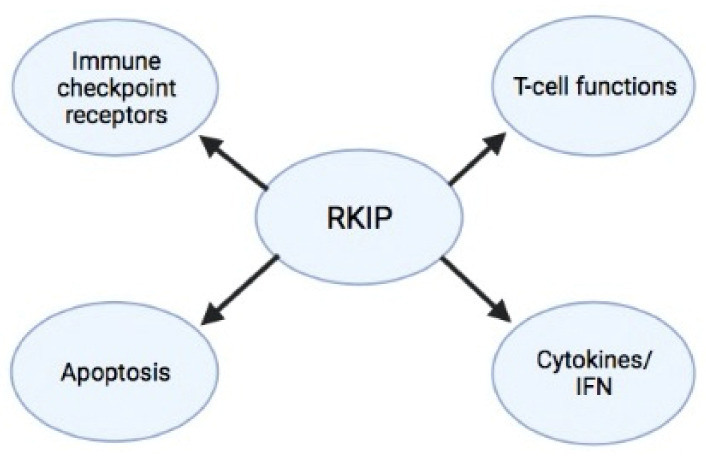
RKIP and immune system cross-talks. RKIP plays a role in the immune system by being in cross-talks with other immune system factors and processes including T-cell functions, cytokines/IFN secretions, apoptosis, and the regulation of immune checkpoint receptors.

Along with the pleiotropic roles of RKIP in regulating distinct intracellular signaling pathways that participate in a wide range of disease pathophysiology, recent findings suggest its involvement in modulating immune-mediated responses in various disease models, including cancer. In particular, RKIP has been reported to contribute to the control of the immune cell infiltration in the tumor microenvironment, in the regulation of host anti-tumor immune-surveillance and responses to immunotherapy, as well as in the modulation of inflammatory processes and the outcome of associated diseases [181]. Below, we discuss how RKIP interferes with and affects the function of immune cells (Figure 3).

### 6.1. RKIP and T Cell Function

The maintenance of immune system homeostasis at ‘immune rest’ or in a state of ‘immune stimulation and action’ is achieved through a sensitive balance of the number, type, localization, and activities of all immune cell populations, and particularly of those involved in adaptive immunity. Any disturbance of this balance can lead to host immune dysfunction. In cancer, the main anti-tumor immune responses are mediated by the tumor antigen recognizing CD8^+^ T cells after their activation and differentiation into effector cytotoxic T lymphocytes (CTLs) by APCs and CD4^+^ T cells [182]. The infiltrated CTLs in the tumor microenvironment eliminate tumor cells by inducing their apoptosis [182]. RKIP has been reported to have direct and indirect effects on CTL-mediated anti-tumor responses via the regulation of pathways involved in CD8^+^ T cell survival, proliferation, differentiation, and cytotoxic activities [182].

It was shown that during metastatic melanoma treatment with dendritic cell (DC) vaccination, there was a correlation between an increase in RKIP expression and an increase in the gene signatures of effective T cell responses, and an inverse correlation with gene signatures of MAPK1 signaling members, Notch1, and STAT3, which are associated with inflammation and myeloid cell infiltration [181,183]. RKIP was also inversely correlated with the myeloid/lymphoid-ratio and was suppressed in patients suffering from chronic inflammatory diseases. This suggests that rather than RKIP being an indicator of a change in immune response towards a productive anti-tumor response, it actually indicated a change in immune response towards chronic inflammation/myeloid immune suppression [181,183]. In addition, it was tested and shown in gastric cardiac adenocarcinoma tissues that little to no RKIP expression often results in low immune function mediated by T-cells and an increase in the metastasis of lymph nodes [125,181]. However, in other models of naïve RKIP deficient mice, the phenotyping of primary and secondary lymphoid organs revealed no underlying deficits in T cell, B cell, NK cell, or CD11b+ APC populations [184].

A decrease in EZH2 expression can cause an increase in RKIP expression, making it possible for the loss of EZH2 expression in cancer cells to be caused by a gain of RKIP expression [98]. An adoptive transfer model was made to get an understanding of the CD8+ T cell-intrinsic role of EZH2 in regard to an antitumoral immune response [185,186]. He et al., transferred Ezh2^fl/fl^Cd4^Cre^ Pmel CD8+ T cells into melanoma B16-tumor-bearing mice and demonstrated the inability of these cells to mediate tumor growth inhibition similar to cells that contained sufficient amounts of EZH2 [185,187]. When transferring Ezh2^fl/fl^Cd4^Cre^ CD8+ T cells into animal subjects, EZH2 deficient T cells were recovered less than control cells and were characterized by reduced IFNγ production, similar to T cells in infection models [185,187,188]. Since researchers were aware that EZH2 function is controlled by phosphorylation, they were able to rescue a significant amount of the defects observed in Ezh2^fl/fl^Cd4^Cre^ CD8^+^ T cells. They did this by either incorporating a phosphorylation insensitive EZH2 or inhibiting the upstream kinase (Akt) [185,187,189]. Other researchers have observed that inhibiting EZH2 activity had beneficial effects, one of them being an increase in RKIP. In Zingg et al.’s murine B16 melanoma model, he displayed that if you combine the EZH2 inhibitor, GSK503, with IL-2 and an anti-IL-2 monoclonal antibody (NARA1) or anti-CTLA-4 therapy, there was a reduction in tumor growth [185,190]. Goswami et al., also demonstrated in a murine bladder model that the combination of EZH2 inhibition and anti-CTLA-4 was able to mediate an even stronger inhibition of tumor growth than respective monotherapies [185,191]. It was found that the antitumor effects were caused by an increase in expression of genes associated with MHC-I peptide processing and increased expression of T cell-recruiting chemokines such as Cxcl9 and Cxcl10 [185,191,192,193]. This deemed EZH2 a relevant target in cancer therapies attempting to improve T cell recruitment into the TME. The effect of EZH2 inhibition on destabilizing the regulatory T cell lineage was demonstrated to impact antitumor immunity [185,191,194]. 

### 6.2. RKIP and Cytokine/Interferon Secretion Patterns

RKIP has been found to play a key role in the production of type I/II interferons by CD8+ T cells after triggering TCR with Staphylococal enterotoxin A (SEA). In the absence of RKIP, effector T cells produce less IFNγ than wild type [195]. In contrast, when the TCR is engaged and combined with TLR stimulation, normal IFNγ levels were synthesized, concluding that by inhibiting RKIP it is possible to halt or decrease the production of IFNγ by effector T cells [195]. It is believed that if researchers were to target RKIP, it could demonstrate a significant benefit over direct IFNγ inhibition, since it alleviates the effects of IFNγ at the synthesis level rather than at the binding of the receptor [195]. However, despite the data suggesting that loss of RKIP leads to a T cell–intrinsic defect in IFN-γ production, the likelihood of T cell–extrinsic effects cannot be excluded.

In 2006, Schuierer et al., showed that RKIP expression may play a role in appropriate macrophage and dendritic cell maturations [195,196]. As the APCs are important for a T-cell activation in response to superantigens and tumor-associated antigens, any dysregulation in the number and functions of APCs could also affect cytokine production from T cells. However, the inhibition of RKIP in APCs had no effect on IFN-γ production, whereas its loss in CD8+ T cells played a critical role on CD8+ T cell-mediated responses to superantigens through a significant reduction in CD8+ T cell-secreted IFN-γ levels [195,196].

### 6.3. RKIP and Apoptosis

Ruiz studied the effects of PKC on T-cell apoptosis related to the signaling of Fas/FasL [122,197]. You can see Fas expression on peripheral CD4+ and CD8+ T cells, some NK cells, and monocytes, while Fas ligand (FasL) is primarily expressed on activated T cells and B cells and even serving as an activated marker for T cells [122]. Generally speaking, when membrane FasL (mFasL) binds with membrane Fas (mFas), the Fas/FasL pathway is activated, which can induce cellular apoptosis. Both the Fas and FasL pathways play a role in regulating lymphocytic quantity in immune responses, which correlates with peripheral activated lymphocyte apoptosis. FasL is highly expressed on activated lymphocytes, along with a high Fas expression [122]. Once they have completed their immune function, activated lymphocytes start to apoptose directly through the Fas/FasL pathway, not allowing the emergence of autoimmune diseases. In the case of a disruption in the Fas/FasL apoptotic pathway, autoimmune disorders are likely to develop.

The activity of NF-kB often makes tumors more resistant to chemotherapy and immunotherapy mediated cytotoxicity by regulating/decreasing the expression of death receptors and expressing anti-apoptotic gene products related to B-cell lymphoma 2 (Bcl-2), 1/2 (c-IAP1/2) proteins, which inhibit apoptosis, the X-linked inhibitor of apoptosis protein (XIAP), and cellular FLICE (FADD-like IL-1β-converting enzyme)-inhibitory protein (c-FLIP) [12,198,199]. Studies showed certain small molecules, such as NPI-0052, a proteasome inhibitor, and DETA/NO, a Nom donor, and immunomodulating agents (e.g., anti-CD20 and anti-CD8) have the ability to sensitize cancer cell lines to chemotherapy related apoptosis and TNF-related apoptosis through inhibition of the NF-kB and Snail signaling and induction of RKIP [12,85,136,200,201,202,203,204]. Furthermore, NF-kB activity is often associated with or the cause of adaptive tumor resistance to treatments, including ionizing radiation [12,205]. Data showing how silencing Snail or RKIP ectopic induction has direct effects that suppress the expression of anti-apoptotic proteins belonging to the Bcl-2 family and the activation of the type II apoptotic pathway supports the conclusion that RKIP and the NF-kB/Snail module have opposing roles in regulating immune and chemoresistance [12,200,202].

Strong evidence points to RKIP having a role in the indirect regulation of the expression of death receptors by means of inhibiting their transcriptional repression, which in turn increases cell sensitivity to immune-mediated cytotoxicity. Death receptors in cancer cells are induced by RKIP because of RKIP’s ability to inhibit the NF-kB/YY1 cascade. YY1 is a multifunctional zinc-finger transcription factor that regulates several cellular functions including, but not limited to, B-cell development, proliferation, tumorigenesis, and differentiation [206,207,208,209]. As a member of the polycomb group (PcG) of proteins, it can work together with other members of the complex [206,207,210,211,212,213]. YY1 represses the transcription of some of these death receptors, including death receptor 5 (DR5) and Fas in different tumor types [12,214,215]. The repression of the transcription of death receptors can be reversed using drugs that promote RKIP expression or RKIP overexpression, which takes away TRAIL tumor resistance and apoptosis mediated by the Fas-ligand [12,90,136,200,216,217,218,219,220,221,222,223,224,225]. Furthermore, sensitizing activities of certain agents involved in immune modulation involved antibody mediated YY1 inhibitions [12,136,218,226,227]. These agents all induce RKIP expression, and RKIP overexpression leads to the suppression of YY1, so it was suggested that the agents tested enhanced sensitizing actions to death by apoptosis through the inhibition of YY1 and death receptor upregulation, both mediated by RKIP.

### 6.4. RKIP and Inhibitory T-Cell Receptors (Immune Checkpoints)

The interaction of activated anti-tumor CD8 T cells with their specific tumor targets results initially in direct killing, but also in their inhibition via the upregulation of inhibitory receptors (eg PD-1. Lag3, TIM) and their interactions with corresponding ligands on the tumor cells [228]. Using a gel shift assay and reporter gene assays, it was confirmed that YY1 binds to the promoters of PD-1 and Lag3 and that there is increased transcription with repeat T-cell stimulation that is caused by a mutation in the YY1 binding sites [206,207,229]. This confirmed that PD-1 and Lag3 are positively regulated by YY1. This was further studied using a luciferase reporter assay that showed an increase in PD-1 in CD8 T cells that contained functional YY1 binding sites as opposed to the decrease or loss of activity when YY1 binding sites were mutated [206,207]. Therefore, RKIP’s inhibition of YY1 leads to a decrease in the expression of PD-1.

In addition, YY1 was identified as a transcription factor for PD-1 and it was found to be highly expressed in melanoma exhausted PD-1^+^ T cells [206,207,230]. This further suggests that the inhibition of YY1 in tumor cells should lead to the inhibition of PD-1 expression in lymphocytes [206,230]. Although tumor-infiltrating lymphocytes (TILs), infiltrate sites when they react to the melanoma antigen Melan-A/MART1, they are often functionally exhausted. This is proven by the low count of cytokines present and the expression of genes, such as PD-1, that are associated with exhaustion [206,207,231]. Fifteen human melanoma samples and 10 normal skin biopsies were examined, confirming the idea that the majority of TILs were exhausted per PD-1 positivity. A study was done where Jun N-terminal kinase (JNK) and p38 mitogen-activated protein kinase (p38 MAPK) phosphorylation was blocked to see how YY1 would be affected, and the results showed that the inhibitors of JNK and p38 MAPK had a suppressive effect on the transcriptional activity of YY1 [206,207]. Once activated, the p38 MAPK/JNK pathway results in the phosphorylation of cJun and activating transcription of ATF2, which in turn promotes transcription and leads to the accumulation of YY1 [206]. These findings revealed that activating the pathway in TILs increases the expression of YY1, which leads to the upregulation of PD-1 and mediates aspects of the exhaustion phenotype. PD-1 expression on CD4^+^ and CD8^+^ T cells in untreated HIV patients is associated with functional exhaustion, with higher fractions of PD-1^+^CD4 T cells [206,207,232,233]. Further studies on the PD-1^+^CD4 T cells confirmed that the PD-1^+^ populations are shifted towards YY1 expression and Ezh2 expression in HIV^+^ subjects [206,207,234].

## 7. Bioinformatics Analyses

**Figure 4:**
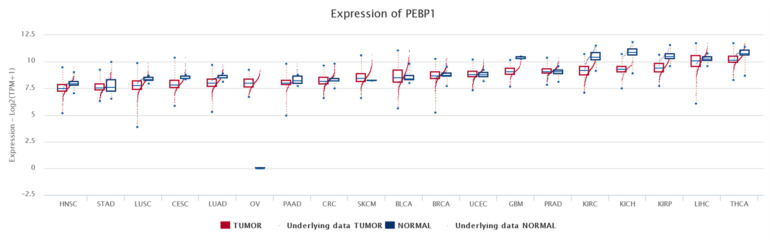
The expression of PEBP1 (log_2_(TPM+1)) across these 19 cancer types in TCGA versus normal tissue. PEBP1 expression was highest in THCA, LIHC, and kidney tumors (KIRP, KICH, KIRC), and significantly lower in several tumor types, such as THCA, KIRP, KICH, GBM, LUAD, CESC, and LUSC, compared to normal tissue.

The Cancer Immunome Atlas (TCIA, https://tcia.at accessed on 29 June 2021) was used to compare the expression of PEBP1 (log_2_(TPM+1)) between cancer and normal tissues across 19 TCGA tumors. PEBP1 expression was highest in THCA, LIHC, and kidney tumors (KIRP, KICH, KIRC). Additionally, PEBP1 was significantly under-expressed in THCA, KIRP, KICH, GBM, LUAD, CESC, and LUSC compared to normal tissue (Figure 4).

**Figure 5:**
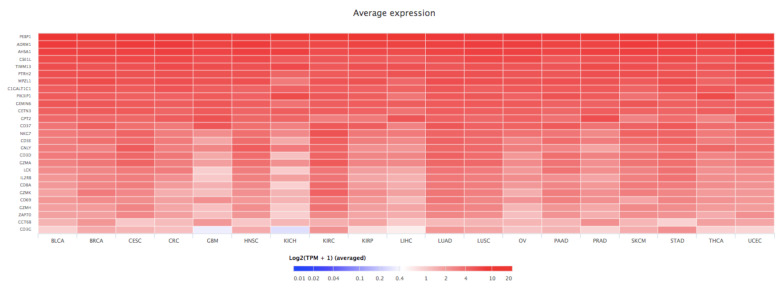
Heatmap depicting the average expression (log2(TPM+1) of CD8+ T cell gene markers along with that of PEBP1 across all 19 TCGA tumors. Genes were ranked based on their expression intensity. PEBP1 was ranked first, followed by ADRM1, AHSA, CSEIL, TRIMM13, and others.

The average expression (log_2_(TPM+1)) of 26 CD8+ T cell gene markers, including CD8A, CD37, CD3D/E/G, IL2RB and NKG7, among others, was also measured along with that of PEBP1 across all 19 TCGA tumors. Specific tumors, including BRCA, CESC, HNSC, KIRC, LUAD, LUSC, PAAD, SKCM and STAD, expressed higher levels of these markers compared to others (e.g., LIHC and KICH); whereas, PEBP1 exhibited the highest expression values (8.62 ± 0.75 log_2_(TPM+1), mean ± SEM) compared to the 26 CD8+ T cell gene markers (Figure 5).

**Figure 6:**
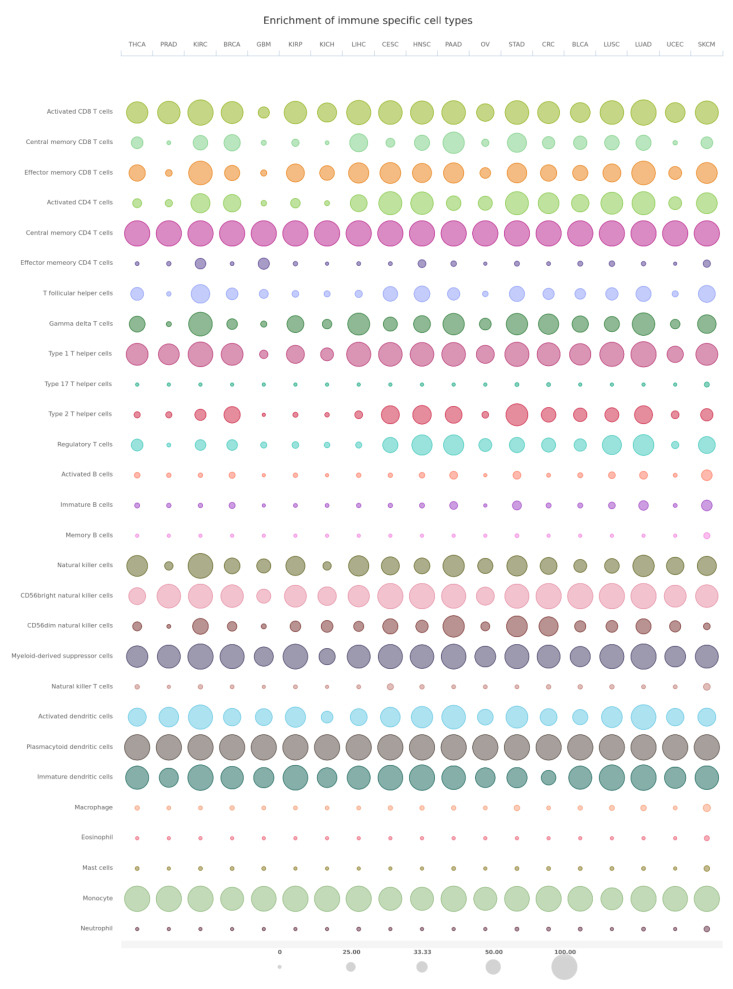
Gene set enrichment analysis (GSEA) plot for 28 different immune specific cells across 19 TCGA tumors. A cutoff of NES > 0 and q-value < 0.1 were considered for the GSEA analysis. Expression of RKIP in activated anti-tumor CD8+ T lymphocytes and regulation—roles in cytotoxicity, survival, and exhaustion.

In addition, gene set enrichment analysis (GSEA) of 28 different immune specific cells across 19 TCGA tumors revealed high enrichment of activated CD8+ T cells, central memory CD4+ T cells, type 1 T helper cells, NK cells, CD56bright NK cells, myeloid-derived suppressor cells, plasmacytoid dendritic cells, immature dendritic cells, and monocytes in the majority of different tumor types (cutoff of NES > 0 and q-value < 0.1) (Figure 6).

## 8. RKIP in Inflammatory Diseases

### 8.1. SIRS

RKIP was suggested as a therapeutic target in the systemic inflammatory response syndrome (SIRS) by decreasing the production of effector cytokines during TCR triggering. It was found that triggering the TCR serves as a good model for human SIRS and reveals IFNγ’s role as a junction between damage from SIRS and a decrease in inflammation in the compensatory anti-inflammatory response syndrome (CARS) [195]. In SIRS, wild-type T cells can produce IFNγ, even if they are unable to produce IL-2, which gives them the ability to enhance the disease. Without RKIP, T cells lose the ability to make enough IFNγ; therefore, its function is critical in these processes [195,235]. Furthermore, inhibiting RKIP using locostatin blocks the production of IFNγ in naïve SIRS T cells [195,235]. To determine the role of RKIP in a SIRS response, cytokine outputs were determined in SIRS and naïve splenocytes. The authors found that T cells in the splenocytes of stimulated mice, compared to naïve controls, produced lower IL-2 levels, implying that T-cells in the culture, similar to the CARS T-cells, were immunosuppressed [195,236]. Further data showed that when SIRS was induced, the percentage of IFNγ that was produced by CD8+ T cells was decreased in RKIP-deficient mice when compared to wild type mice (Table 2). Of the CD8+ T cells that produce IFNγ, wild type cells produced higher amounts when compared to RKIP-deficient cells. It was noted that this defect was not observed in staphylococcal enterotoxin A (SEA)-specific CD4+ T cells, bystander T cells, or in splenic non-T cell populations [195]. Naïve T cells also did not produce IFNγ, which suggests that poor production in SIRS splenocytes lacking RKIP is due to a defect in the CD8+ T cells. RKIP is a potentially valuable therapeutic target for SIRS since by inhibiting RKIP, IFNγ synthesis is stopped without shutting down responses to pathogen-associated molecular patterns (PAMPs). RKIP has been correlated with IFNγ potential in immunosuppressed or anergic CD8+ T cells [195,237] and is also a negative regulator of the NF-kB and MAPK pathways, both of which are essential for producing proinflammatory cytokines [195,238,239]. It does so by targeting the activities of kinases belonging to signaling factors in these pathways and interfering with them. The findings from the study explained above showed that contrary to their hypothesis, RKIP deficient mice would not experience inflated cytokine production during the activation of T cells, scientists found that enhanced production of IFNγ in CD8+ SIRS T cells could only happen if RKIP was present [195,240]. In addition, they conclude that RKIP has a role in the downstream signaling of the TCR, which they were able to do because of data that showed that using PMA and ionomycin to bypass the TCR actually reversed the reduction in IFNγ production from RKIP deficient CD8+ T cells [195]. In a SIRS model, it was found that MAPK-mediated expansion of T cells was unaltered, which implies that in effector T cells, RKIP has a more cardinal role for cytokine output as opposed to proliferation [195,241].

Wright and his team ran an experiment where they restimulated splenocytes from wild type mice 48 h after they were induced with SIRS, SEA, and PMA+ ionomycin with locostatin present in order to test blocking RKIP for therapeutic purposes. Similar to what was seen in RKIP deficient SIRS splenocytes, inhibiting RKIP using locostatin led to a reduction in IFNγ production significantly after T cell receptors were retriggered with SEA, but were not reduced after restimulation with PMA+ ionomycin, which, again, suggests that downstream of the TCR, RKIP has a role [242]. All in all, it was demonstrated that during a SIRS response, CD8+ T cells may cause cytokine production, and RKIP is correlated with continued IFNγ potential in CD8+ T cells that are immunosuppressed or anergic [238,239,242,243,244,245].

### 8.2. AITP

Acute idiopathic thrombocytopenic purpura (AITP) is an autoimmune disease common in children and younger females. Studies showed that PKC is an important enzymatic component of the underlying mechanism of this disease development through its role in cell signal transduction and T cell proliferation and apoptosis [122,197,246] (Table 2). PKC is expressed on T lymphocytes and is required for TCR-triggered differentiation of naive T cells into mature (effector) T cells. The association of T cells with antigen-presenting cells (APCs) often results in the recruitment of PKC to T cell-APC contact areas, where it induces activation signals by interacting with signal molecules [122]. PKC also plays a role in promoting T cell cycle progression and proliferation [122,247]. In a study by Wu et al., (2005), they found that in children with immune thrombocytopenic purpura (ITP), PKC activity was enhanced and there was a positive correlation between PKC activity and the expression of FASL in T cells [122]. They also found that T cells were hypersensitive to PMA stimulation in ITP children in comparison to healthy children, resulting in more T cell activation and thrombocyte damage [122].

PKC expression levels and activity have been associated with a number of autoimmune diseases. PKC activity was found to be elevated in the fibroblasts of type 1 diabetic patients, while decreased PKC activity in patients with systemic lupus erythematosus (SLE) was correlated with the state of their condition [122,248,249].

An increase in the expression of PKC leads to an increase in the activation of RKIP, which is why the former is thought to be a good target for cancer therapy. It is expressed on T lymphocytes and is necessary for TCR-triggered activation of mature T cells. In order for proper T cell activation and IL-2 production to occur, engagement of T cells by APCs must occur because that results in the recruitment of PKC to T cell-APC contact areas, where it induces activation signals by interacting with signal molecule signals [122], one of those signaling pathways being that of RKIP. PKC also plays a role in promoting T cell cycle progression and proliferation [122,247]. These findings suggested that PKC can be used as a drug target for immunosuppression in ITP and in other auto-immune diseases.

## 9. Discussion and Perspectives

In this review, we have described the pleiotropic activities of RKIP expression in cancer and immune cells and different examples of inflammatory immune diseases. Clearly, the levels expressed dictate the outcomes. For instance, the low expression RKIP is associated with tumor progression in many cancers, metastases and resistance to cell death by chemo-immuno-therapeutic drugs. Conversely, the induction of RKIP expression reverts these manifestations, inhibits tumor growth and metastases, and sensitizes the tumor cells to cell death by chemo-immunotherapeutic drugs. Also, RKIP expression in T cells regulates immune inflammatory diseases through various mechanisms, including the induction of IFNγ secretion. In both instances, it is clear that RKIP is a potential therapeutic target through its induction in cancers and its inhibition in inflammatory diseases [62,250].

## 10. Induction of RKIP 

**Figure 7:**
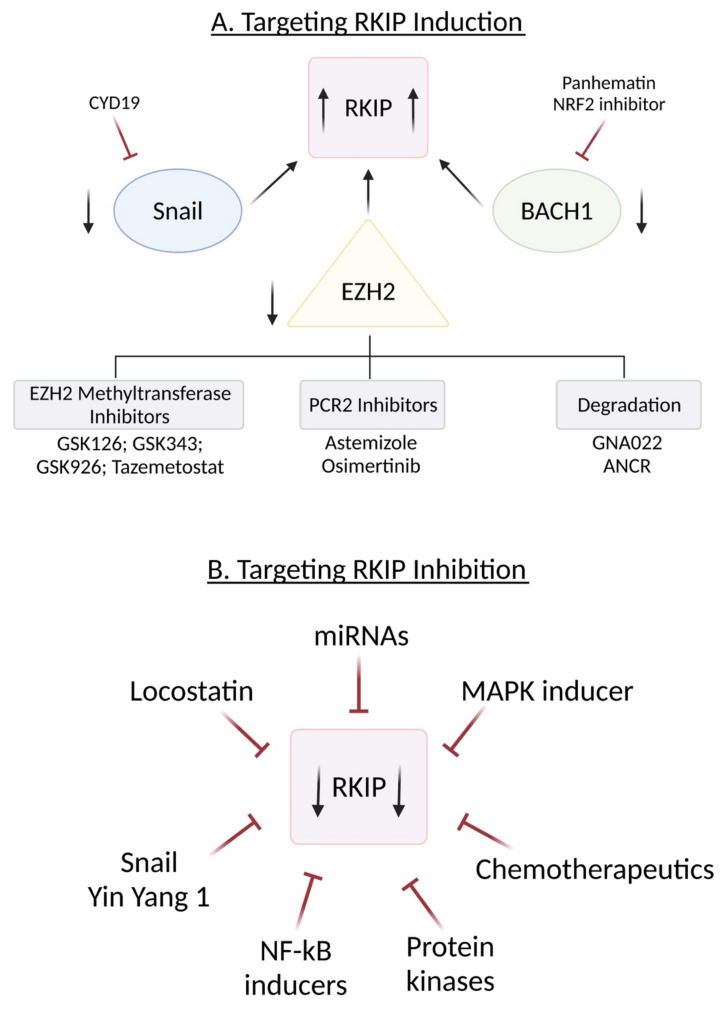
(**A**). Targeting RKIP induction. Targeting RKIP expression in various cancers results in the inhibition of cell proliferation, invasion, and resistance to cytotoxic agents. Several means have been reported to induce the expression of RKIP. These include inhibiting the RKIP repressor SNAIL via a small molecule, CYD19, that leads to SNAIL degradation. Targeting the RKIP suppressor BACH1 via small inhibitors such as hemin or the FDA approved Panhematin as well as NRF2 inhibitors will lead to upregulation of RKIP expression. Targeting EZH2 also results in the upregulation of RKIP expression. This can be achieved by many ways including (i) EZH2 methyltransferase inhibitors (GSK126; GSK343; GSK926; Tazemetostat) (ii) PCR2 inhibitors (Astemizole, Osimertinib) and (iii) Degradation (GN022, ANCR). (**B**) Targeting RKIP Inhibition. Locostatin interacts with the RKIP ligand-binding pocket and is the main inhibitor of RKIP. The induction of the RKIP suppressor SNAIL or its regulator Yin Yang 1 (YY1) will inhibit RKIP. The activation of NF-KB, MAPK, and protein kinases will inhibit and/or phosphorylate RKIP. In addition, various chemotherapeutic drugs and specific miRNAs can be used to inhibit RKIP expression.

The induction of the repressed-RKIP in cancer to reverse the oncogenesis, metastasis and resistance-mediated effects may be achieved by various means that can lead to its induction. For instance, the major repressors, SNAIL and BACH 1 or EZH2, may be targeted for their inhibitions. Briefly, the various means by which these repressors can be targeted, are described below (Figure 7A).

### 10.1. Targeting SNAIL

The zinc finger transcription factor Snail is aberrantly activated in a variety of different human cancers and associated with poor prognosis [251]. It also plays an essential role in EMT, metastasis, stem cell–like properties, cancer metabolism, modulation of the micro-environment, immune evasion, cancer recurrence, and therapeutic resistance. Snail is a repressor of RKIP [251]. Therefore, SNAIL targeting will result in the de-repressing of RKIP and the inhibition of tumor growth and metastasis. Li et al., (2020) recently reported that the small molecule, CYD19, binds with high affinity to SNAIL, resulting in the disruption of the interaction between SNAIL and the CRB-binding protein (CBP)/p300 [251]. This disruption prevents the acetylation of SNAIL, leading to its degradation. In addition, CYD19 restores the SNAIL-dependent suppression of p53. Altogether, CYD19 treatment inhibits tumor growth, invasion, and EMT, and restores other RKIP-mediated activities, as well as chemo-immuno-sensitizing activities [251].

### 10.2. Targeting BACH1

Bach1 suppresses RKIP, but it is also suppressed by it [252]. Bach1 also self-represses by binding to its promoter, thus creating its own negative feedback loop [92]. The direct inhibition of BACH1, a repressor of RKIP, may be targeted for the induction of RKIP. BACH1 and BACH2, two BACH family protein members, have homologous sequences and structures containing a BTB and the bZIP domain, heme-binding motifs, and the cytoplasmic localization signal (CLS) [253]. In particular, BACH1 blockade through shRNA, sgRNA, or small inhibitors such as hemin (an iron-containing porphyrin from a heme group [254]), reduced its expression in cancer cells and was sufficient to efficiently inhibit metastasis successfully and efficiently. This suggested that hemin may be a pharmacological inhibitor. The injection of hemin is currently available in the form of the FDA-approved non-toxic BACH1-specific drug, Panhematin, which is used to treat patients with acute porphyria [255]. Hemin treatment in tumors showed a significant decrease in BACH1 levels and a change in metabolic pathways.

In addition to the above inhibitors of BACH1, BACH 1 competes with nuclear factor (erythroid derived 2)-like 2 (NRF2) for binding to the MAREs in oxidative stress response genes [256] and subsequently BACH1 exported out of the nucleus into the cytoplasm [252]. In a study by Lignitto et al., Nrf2 accumulated in lung cancers and caused the stabilization of Bach1 by inducing Ho1, the enzyme catabolizing heme [257]. Therefore, NRF2 inhibitors will also inhibit BACH1, as previously reviewed by Robledinos-Anton et al., (2019), and are still far from being translated from bench to bedside [258].

### 10.3. Targeting EZH2

EZH2 alters gene expression and regulates cell cycle progression by trimethylating Lys-27 in histone 3 (H3K27me3) [259]. Its dysregulation was shown to accelerate cell proliferation and increase cell survival, driving carcinogenesis [260].

The inhibition of EZH2 was shown to induce RKIP [98]. Duan et al., (2020) discussed several EZH2 methyltransferase inhibitors, which break the structure of the polycomb repressive complex 2 (PRC2), of which EZH2 is a catalytic subunit, as well as inhibitors that suppress EZH2 via triggering its degradation, or a combination of inhibitors with different treatment options [261]. Hence, such inhibitors will result in the induction of RKIP expression. Briefly these are presented below.

(1) Inhibitors of EZH-2 methyltransferase activity.

During the last decade, there has been a plethora of inhibitors of the EZH2 methyltransferase activity, most of which occupy the binding pocket of the protein of the co-substrate S-adenosyl-methionine (SAM). GSK126 (GSK2816126) is highly selective and has the ability to inhibit Y641 mutant EZH2 and wild type EZH2 with similar potency, even though SAM is known to be the methyl donor [262]. In addition, SAM-competitive inhibitors of EZH-2 have been developed, such asGSK343, GSK926, and tazemetostat (E7438/EPZ6438). Tazemetostat can be delivered orally and has better pharmacokinetic properties and potency [263]. Recently, tazemetostat (TAZVERIK, Epizyme, Inc., Cambridge, MA, USA) received FDA approval for use by pediatric patients ≥16 years old and adults that have metastatic or locally advanced epithelioid sarcoma and are not eligible or approved for complete resection. In addition, CPI-1205, an orally bioavailable, indole-based, small-molecule inhibitor of EZH2 was evaluated in a B cell lymphoma phase 1 clinical trial (NCT02395601) [264].

(2) Inhibitors that break PCR2 structure

One way to inhibit EZH2 is to disrupt the protein–protein interactions within the PRC2 subunits. Astemizole is approved by the FDA and is an H1 histamine receptor antagonist that disrupts the EZH2-EED complex in order to stop PRC2-driven lymphoma cells from proliferating [265]. In addition, AZD9291 (Osimertinib, TAGRISSO; USA, DE) inhibits EGFR and is FDA approved for treating patients that have been diagnosed with EGFR^T790M^ metastatic NSCLC. This drug functions by breaking the structure of EZH2-EED [266]. Other inhibitors were recently reviewed [261].

(3) Triggering EZH2 degradation

The gambogenic acid (GNA) derivative, GNA022, was reported to decrease the stability of the PRC2 complex and H3K27 trimethylation, which would result in the triggering of ubiquitination mediated EZH2 degradation [267]. Furthermore, ANCR, the long non-coding RNA (lncRNA), can promote the CDK1–EZH2 interaction, resulting in EZH2 ubiquitination and its degradation in breast cancer cells in vitro. In mice with breast cancer, ANCR was shown to repress distant metastasis and tumor growth [268].

(4) EZH2 inhibitors combined with other therapies

The combination of the anti-CTLA-4 immunotherapeutic drug, ipilimumab, with CPI-1205 was evaluated in a phase 1/2 study (NCT03525795) in patients with advanced solid tumors. Researchers demonstrated the combination between conventional chemotherapy and EZH2. Additionally, synergistic effects of tazemetostat with drugs such as prednisone, oncovin, doxorubicin, and cyclophosphamide were observed in EZH2-mutant DLBCL tumors [263].

## 11. Inhibition of RKIP 

For the inhibition of RKIP, one may target the above gene products. In addition, Shemon et al., (2009) reported that locostatin interacts with the RKIP ligand-binding pocket [29]. Treatment with locostatin induced T cell energy by blocking cytokine production [269]. This was associated with a reduction in ERK phosphorylation.

The various interactions of RKIP with the Raf-1/MEK/ERK, NF-kB pathways, and inflammatory fatty acids/phospholipids strongly suggest its role in various inflammatory diseases (Figure 7B). These interactions may be associated with both unphosphorylated and phosphorylated RKIP. The PKC-mediated phosphorylation of RKIP at the Ser153 residue induces a conformational change in the ligand binding pocket, contributing to the dissociation of RKIP from Raf-1 and leading to the activation of ERK/MAPK pathways. ERK has the ability to induce multiple regulatory genes that regulate inflammatory responses. Although ERK does not stimulate gene expression directly and by itself, it does so by modulating signaling pathways such as NF-κB, PI3K, and interferon-regulatory factor (IRF) transcription factors. Accordingly, the MEK/ERK pathway is recognized by researchers as a critical pathway that is typically pro-inflammatory [270].

Lin et al., (2018) reported that RKIP is involved in autoimmune inflammation through the regulation of IL-17R signaling [271]. Th17 cells contribute to the development of autoimmune diseases via the secretion of IL-17. RKIP deficiency in mice improves the symptoms of EAE. RKIP promotes IL-17R mediated pro-inflammatory cytokine and chemokine production. These authors reported that RKIP interacts directly with IL-17RA and Act1, leading to the formation of an IL–17RA–Act1 complex. This complex enhances the MAPK and NF-kB activations and downstream cytokine production.

Overall, the above findings illustrate that the level of expression of RKIP in cancer and immune cells mediating inflammatory diseases dictate the outcome. Since RKIP is a pivotal factor in these regulatory mechanisms, the search for agents that can be specific and target RKIP (for induction or suppression) may be useful therapeutically. The current investigations, both pre-clinically and clinically, are geared towards the development of RKIP-specific therapeutic agents.

## Figures and Tables

**Table 1:** RKIP expression in various cancers and their functions.

	Expression of RKIP	Impact/Functions	References
Adenocarcinomas	Decreased RKIP expression	RKIP increases progression, metastasis, and invasion leading to a poor prognosis	Wei et al., 2015; Wei et al., 2014
Colon Cancer	Reduction of RKIP	Amplifies radio-resistance and chemoresistance	Zaravinos et al., 2018; Lee et al., 2016
Prostate Cancer	RKIP is downregulated	Enhances metastasis in prostate cancer-associated cell lines	Beach et al., 2008
Pancreatic Cancer	RKIP is induced	Prevents the invasive metastasis of pancreatic cancer cells	Kim and Kim, 2012
Gliomas	Low expression of RKIP	Does not affect cell proliferation, and enhances cell migration	Martinho et al., 2012
Renal Cell Carcinoma	High RKIP expression	Induces cell survival and progression-free survival	Papale et al., 2017
Gastric Cancer	Low levels of RKIP expression	Negatively correlated with depth of tumor invasion	Wang et al., 2010
Lung Cancer	Decreased levels in invasive cancers	Greater advantage in survival	Huerta-Yepez et al., 2011
Leukemia	Loss of RKIP expression is common	RKIP inhibits proliferation of myeloid cells	Zebisch et al., 2019
Multiple Myeloma	RKIP is overexpressed	Enhances tumor progression	Shvartsur et al., 2017

**Table 2:** The role of RKIP in inflammatory diseases.

	Expression of RKIP	Impact/Functions	References
SIRS (systemic inflammatory response syndrome)	RKIP expression is decreased	IFNy production is decreased leading to induction of SIRS	Wright and Vella, 2013
AITP (acute idiopathic thrombocytopenic purpura)	RKIP activation is increased (when PKC expression increases)	Increases the functions of T cells progression and proliferation	Wu et al., 2005

## Data Availability

Not applicable.

## Abbreviations

ADT	androgen deprivation therapy
APCs	antigen-presenting cells
AR	androgen receptor
ARE	androgen responsive element
B-AR	beta-adrenergic receptor
BACH1	BTB and CNC homology 1
Bcl-2	B-cell lymphoma 2
Bp	base pairs
c-FLIP	FLICE-inhibitory protein
cAMP	cyclic adenosine monophosphate
CD	cluster of differentiation
CDK5	cyclin dependent kinase 5
ceRNA	competing endogenous RNA
CREB	cyclic adenosine monophosphate response element-binding protein
DC	dendritic cell
Dlg	discs-large tumor suppressor
DR5	death receptor 5
E-cad	E-cadherin
EGCG	epigallocatechin 3-gallate
EMT	epithelial-to-mesenchymal transition
EZH2	enhancer of Zeste homolog 2
FasL	Fas ligand
FLICE	FADD-like IL-1β-converting enzyme
GPCRs	G-protein-coupled receptors
GRK2	G protein-coupled receptor kinase 2
GRK2	GPCR kinase 2
GSK3β	Glycogen synthase kinase 3
HDAC	histone deacetylase
HHC	human hepatoma
HSC	hepatic stellate cells
IKK	IkB kinase
ITP	immune thrombocytopenic purpura
JAK 1/2	Janus kinase 1/2
JNK	Jun N-terminal kinase
lncRNA	long non-coding RNA
LPS	lipopolysaccharide
MAPK	MAP kinase
MDA-9	melanoma differentiation associated gene 9
mFas	membrane Fas
mFasL	membrane FasL
MREs	miRNA recognition elements
NIK	NF-kB-inducing kinase
NPC	nasopharyngeal carcinoma
NSCLC	non-small-cell lung cancer
PCa	prostate cancer-associated
PcG	polycomb group
PEBP	phosphatidylethanolamine-binding protein
PKC	protein kinase C
PRC2	polycomb repressive complex 2
PSD-95	postsynaptic density protein
RKIP	Raf kinase inhibitory protein
RTKs	receptor tyrosine kinases
S153	Serine 153
SEA	staphylococcal enterotoxin A
SLE	systemic lupus erythematosus
Sp1	specificity protein 1
STAT	signal transducer and activator of transcription
TAK-1	transforming growth factor B-activated kinase-1
TCR	T cell antigen receptor
TILs	Tumor-infiltrating lymphocytes
TNBC	triple-negative breast cancer
TSA	trichostatin A
XIAP	X-linked inhibitor of apoptosis protein
XIST	X-inactive specific transcript
YY1	Yin Yang 1
ZO-1	tight junction protein-1
